# Diagnosis of complication in lung transplantation by TBLB + ROSE + mNGS

**DOI:** 10.1515/med-2020-0232

**Published:** 2020-10-01

**Authors:** Qing Wang, Jing Feng, Ji Zhang, Lingzhi Shi, Zhixian Jin, Dong Liu, Bo Wu, Jingyu Chen

**Affiliations:** Respiratory Department of Kunming Municipal First People’s Hospital, Kunming 650000, China; Graduate School, Tianjin Medical University General Hospital, Tianjin 300052, China; Respiratory Department of Tianjin Medical University General Hospital, Tianjin 300052, China; Respiratory Department of Lung Transplant Center, The Affiliated Wuxi People’s Hospital of Nanjing Medical University, Wuxi 214023, China

**Keywords:** lung transplantation, infection, complication, transbronchial lung biopsy, rapid on-site cytological evaluation, metagenomic next-generation sequencing

## Abstract

Lung transplantation is a potentially life-saving therapy for patients with terminal respiratory illnesses. Long-term survival is limited by the development of a variety of opportunistic infections and rejection. Optimal means of differential diagnosis of infection and rejection have not been established. With these challenges in mind, we tried to use transbronchial lung biopsy (TBLB) rapid on-site cytological evaluation (ROSE), metagenomic next-generation sequencing (mNGS), and routine histologic examination to timely distinguish infection and rejection, and accurately detect etiologic pathogens. We reviewed the medical records of all patients diagnosed with infection or rejection by these means from December 2017 to September 2018 in our center. We identified seven recipients whose clinical course was complicated by infection or rejection. Three patients were diagnosed with acute rejection, organizing pneumonia, and acute fibrinoid organizing pneumonia, respectively. Four of the seven patients were diagnosed with infections, including *Pneumocystis carinii* pneumonia, cytomegalovirus, *Aspergillus*, and bacterial pneumonia. These patients recovered after proper treatment. TBLB + ROSE + mNGS might be a good method to accurately detect etiologic pathogens, which may help us to facilitate the use of targeted and precision medicine therapy in postoperative complications and avoid unnecessary potential adverse effects of drugs.

## 1 Introduction

Lung transplantation is a potentially life-saving therapy for patients with terminal respiratory illnesses that are refractory to conventional therapies such as idiopathic pulmonary fibrosis (IPF), emphysema, cystic fibrosis, bronchiectasis, sarcoidosis, pulmonary arterial hypertension, lymphangioleiomyomatosis, and so on [1].

The estimated posttransplant long-term survival rate is 50% at 5 years and 27% at 10 years [2], long-term survival is limited by the development of a variety of opportunistic infections, acute allograft rejection, and chronic lung allograft dysfunction [3]. According to the latest report from the International Society for Heart and Lung Transplantation, 29% of adult patients have at least one episode of treated acute rejection during discharge from the hospital and 1 year follow-up after transplant [4]. The widespread use of prophylactic antibiotics and various techniques to monitor pulmonary complications has helped to decrease infectious pulmonary complications in patients who undergo lung transplantation.

Early recognition of the signs of complications that distinguish rejection from infection allows treatment to be initiated earlier, which may improve clinical outcomes for lung allograft recipients. But the clinical and radiological manifestations of postoperative complications can be non-specific and at times confusing, such as acute transplant rejection is characterized clinically by rapid onset of fever, infiltrates on computed tomography (CT) scan, and decreased gas exchange which can also be seen in the condition of infection. Since it is difficult to rule out infectious pulmonary complications by manifestations and CT scan findings, it is often unavoidable to start empiric therapy based on clinical and radiologic findings without a definitive diagnosis by pathological or microbiological findings, which is of uncertain accuracy [5]. This approach can lead to inappropriate treatment with the ensuing risks of possible adverse events, while potentially reversible causes may go unrecognized.

In these situations, further bronchoscopy usually tried to make a definite diagnosis. The specimens obtained by transbronchial lung biopsy (TBLB) are usually fixed in formalin and embedded in paraffin for histologic examination [6]. Supplementary methods such as rapid on-site cytological evaluation (ROSE) and metagenomic next-generation sequencing (mNGS) also are used to supply some cytological and microbiological information, respectively.

In 2017, we developed the protocol ROSE for recognition of complications, which may be useful to distinguish rejection from infection to some extent. ROSE of biopsy allows the bronchoscopist and pathologist to assess whether the specimen is adequate and diagnostic, and triage material for ancillary testing, including immunohistochemistry, predictive molecular testing, flow cytometry, microbiologic cultures, and special stains. It can avoid unnecessary delays and allow better specimen acquisition [7]. ROSE also provide a preliminary diagnosis for clinicians, while information from ROSE cannot replace pathological or microbiological analyses, it may provide extra information that aids decision making for clinicians weighing treatment options in cases of borderline rejection and infection. What’s more, results of ROSE are available before pathological interpretation of transbronchial biopsies and microbiologic cultures are complete, these predictors could be useful in cases when patients present with a clinical syndrome consistent with either infection or rejection.

The detection of pathogens is a prerequisite to exclude infections. mNGS also called high-throughput sequencing, allows for unbiased detection of virtually any pathogen present in a given sample [7,8,9,10]. In thisstudy, we report several cases and reviewed our institutional experience for diagnosing infection and rejection based on clinical, radiologic, cytologic, pathological, or microbiological findings. Especially, we focused on the use of TBLB + ROSE + mNGS for recognition of postoperative complications of lung transplantation.

## 2 Methods

### 2.1 Study subjects

This study was approved by the ethics board of our institution. All subjects gave written informed consent. All patients who received lung transplants at Wuxi People’s Hospital and had consecutive surveillance or clinically indicated bronchoscopy with TBLB and ROSE and/or mNGS and histologic examination from December 2017 to September 2018 were included. Data collection included age, gender, underlying conditions, indication for transplant, single versus bilateral transplant, maintenance immunosuppression, symptoms, radiographic and histopathologic findings, pulmonary function testing, routine microbial testing, bronchoscopy, ROSE and mNGS information, treatment methods, and follow-up outcome.

### 2.2 Postoperative immunosuppression

The immunosuppressive regimen after transplantation was similar to that used by other major transplant centers. Maintenance triple immunotherapy in our center included cyclosporine or tacrolimus, mycophenolate mofetil, and corticosteroids.

### 2.3 Bronchoscopy

Surveillance bronchoscopy was taken at postoperative months 1, 3, 6, 9, 12, 18, and 24. Clinically indicated bronchoscopy was performed in patients with suspected rejection or infection based on symptoms such as fever, dyspnea on effort, hypoxemia, diagnostic imaging, pulmonary function testing, or blood laboratory data. After written informed consent was obtained, bronchoscopy was performed including bronchoalveolar lavage (BAL), brushing, and TBLB in all cases. On each patient, TBLB was performed at first, meanwhile, ROSE was performed during the examination to determine whether the sample was sufficient for diagnosis. Then brushing was performed using a protected-specimen brush and bronchoalveolar lavage fluid (BALF) specimens were obtained after brushing.

### 2.4 ROSE

Biopsy specimens were expressed onto labeled glass slides and smearing was performed by an on-site cytotechnologist. Rapid staining was performed using Diff-Quik stain. Cytologic glass slides were then evaluated under light microscopy by the cytotechnologists for immediate interpretation of whether the sample was sufficient for a provisional diagnosis as well as for all later laboratory requirements. Quick feedback was sent back to the bronchoscopist by cytotechnologists. The bronchoscopist terminated or modified the sampling process based on the information. If the diagnostic objective has been met from imprint cytology, sampling was stopped, conversely, if no provisional diagnosis resulted from imprint cytology, sampling was continued with the appropriate modalities.

### 2.5 Routine histologic examination

After smearing was performed, the remaining material was placed in 10% formaldehyde and embedded in paraffin for routine histologic examination using cell block preparation. Five serial sections of each cell block were stained with hematoxylin and eosin, Masson trichrome for lung architecture, orcein for vessel identification, and hexamine silver for fungi and *Pneumocystis carinii*.

### 2.6 Microbiological study

#### 2.6.1 Routine microbial testing

BALF was used for microbial culture for bacteria, mycobacteria, and fungi. Besides culture, other traditional pathogen detection methods based on invasive respiratory specimens obtained through bronchoscopy are as follows, the TBLB specimens were sent to the histopathology laboratory for pathological examination. They were stained with hematoxylin and eosin to examine inflammatory cell infiltration and the presence of visible pathogens (fungal hyphae). TBLB specimens also underwent special pathological staining, including acid-fast staining and hexamine silver staining. The protected-specimen brush was smeared evenly on a slide and was sent to the histopathology laboratory for acid-fast staining. The BALF sent to the microbiology laboratory to underway Galactomannan antigen detection (GM test), *Mycobacterium tuberculosis*/rifampicin-resistance test (X-pert), and centrifugal sediment of BALF were smeared on slides for gram staining, acid-fast staining, and hexamine silver staining. Gram stain, GM test, hexamine silver staining, X-pert, and acid-fast stain were used to identify bacteria, fungi, *Pneumocystis carinii*, and *Mycobacterium tuberculosis* under a microscope. Available serological examinations include nucleic acid detection through PCR testing for common respiratory viruses (epstein-barr virus, human cytomegalovirus [CMV], respiratory syncytial virus, parainfluenza virus, and adenovirus), serum cryptococcal capsular polysaccharide antigen detection for *Cryptococcus neoformans* and *Mycoplasma pneumoniae* antibodies detection.

#### 2.6.2 mNGS

Sample processing and nucleic acid extraction: lung biopsy specimen was collected and cut into small pieces. Samples of 0.5–3 mL BALF were collected from patients following standard procedures. DNA was extracted using the TIANamp Micro DNA Kit (DP316, TIANGEN BIOTECH) according to the manufacturer’s recommendation. Construction of DNA libraries: single-stranded DNA circle library was constructed after DNA-fragmentation, end-repair, adapter-ligation, DNA denaturation into single strands, DNA circularization. DNA nanoballs were generated from the single-stranded DNA circle using rolling circle amplification. Finally, qualified DNA nanoballs were loaded on the flow cell and sequenced on BGISEQ-50 platform. Sequencing and bioinformatic analysis: high-quality sequencing data were generated by removing low-quality, and short (length <35 bp) reads, followed by computational subtraction of human host sequences mapped to the human reference genome (hg19) using Burrows–Wheeler alignment. After the removal of low-complexity reads, the remaining data were classified aligning to four Microbial Genome Databases simultaneously, consisting of viruses, bacteria, fungi, and parasites. The databases were downloaded from NCBI (ftp://ftp.ncbi.nlm.nih.gov/genomes/). It contains 4,061 viral taxa whole genome sequence, 2,473 bacterial genomes or scaffolds, 199 fungi related to human infection, and 135 parasites associated with human diseases.

### 2.7 Diagnosing strategies of rejection and infection

For diagnosis of allograft rejection, tissue diagnosis is necessary. Obtaining lung tissue through TBLB is currently the gold standard to assess patients for lung allograft rejection and to distinguish rejection from other clinical mimics such as infection, drug toxicity, and recurrent disease. The “International Society for Heart and Lung Transplantation” published a revision of the “working formulation for the standardization of nomenclature in the diagnosis of lung rejection” in 2007 [11], which established the diagnostic criteria for rejection. The final diagnosis of pulmonary infections is confirmed by a comprehensive analysis of clinical manifestation, imaging manifestation, findings of traditional pathogen detection based on respiratory specimens mentioned above, mNGS, serological examination, ROSE, and histopathology, expert opinion, and treatment effect observations.

## 3 Results

We reviewed seven postoperative patients of lung transplantation who had clinically indicated bronchoscopy with TBLB and ROSE and mNGS and histologic examination from December 2017 to August 2018. Demographic and clinical information of these seven patients is given in Table 1. Routine culture, mNGS, ROSE, and histologic examination findings are shown in Table 2. A series of cases are displayed as follows.

**Table 1:** Demographic and clinical information of the 7 patients

Cases	Age (years)	Gender	Transplant indication	Other underlying conditions	Transplant type	Time onset (days post-transplant)	Diagnostic modality	Complication type
Case 1	33	Male	PH	No	BLT	251	TBLB	CMV infection
Case 2	38	Male	IPPEE	No	BLT	599	TBLB	*Aspergillus* infection
Case 3	28	Male	GVHD	Diffuse large B cell lymphoma	BLT	314	TBLB	PCP
Case 4	52	Male	IPF	PH, PHD	SLT (right)	45	TBLB	Infection
Case 5	56	Male	IPF	Hypertension	SLT (left)	74	TBLB	AFOP
Case 6	45	Male	Congenital bronchiectasis	No	BLT	373	TBLB	OP
Case 7	73	Female	SPF	SS, Hypertension	SLT (right)	126	TBLB	Acute rejection

PH: pulmonary hypertension; BLT: bilateral lung transplant; TBLB: transbronchial lung biopsy; CMV: cytomegalovirus; IPPFE: idiopathic pleuroparenchymal fibroelastosis; GVHD: Graft-Versus-Host Disease; PCP: *pneumocystis carinii* pneumonia; IPF: idiopathic pulmonary fibrosis; PHD: pulmonary heart disease; SLT: single lung transplant; OP: organizing pneumonia; SPF: secondary pulmonary interstitial fibrosis; SS: sjogren syndrome.

**Table 2:** Routine culture, mNGS, ROSE, and histologic examination findings

Test				
Cases	Routine culture	mNGS	ROSE	Histologic examination
Case 1	Negative	CMV and *Pseudomonas*	Intranuclear inclusion body of the virus, infiltrates of lymphocytes, and increased alveolar macrophages	Intranuclear inclusion body of the virus, immunohistochemistry shows CMV+
Case 2	Negative	*Aspergillus*	Typical septate hyphae with dichotomous branches	Histologic examination failed to find typical septate hyphae, negative methenamine silver staining
Case 3	Negative	*Pneumocystis carinii*	Infiltrates of lymphocytes, increased alveolar macrophages	Alveolar septum is infiltrated of a few lymphocytes, neutrophils, exfoliated alveolar epithelial cells
Case 4	Negative	Miscellaneous bacteria	Infiltrates of neutrophils	Infiltrates of large number of neutrophils and a few lymphocytes
Case 5	Negative	Negative	Infiltration of lymphoid cells and large number of foamed macrophages and fibroblasts	AFOP
Case 6	Negative	Negative	Conform to organization	Organizing pneumonia
Case 7	Negative	Negative	Infiltrates of lymphocytes and macrophages	Minimal acute rejection

NGS: next-generation sequencing; ROSE: rapid on-site cytological evaluation; CMV: cytomegalovirus.

**Figure 1:**
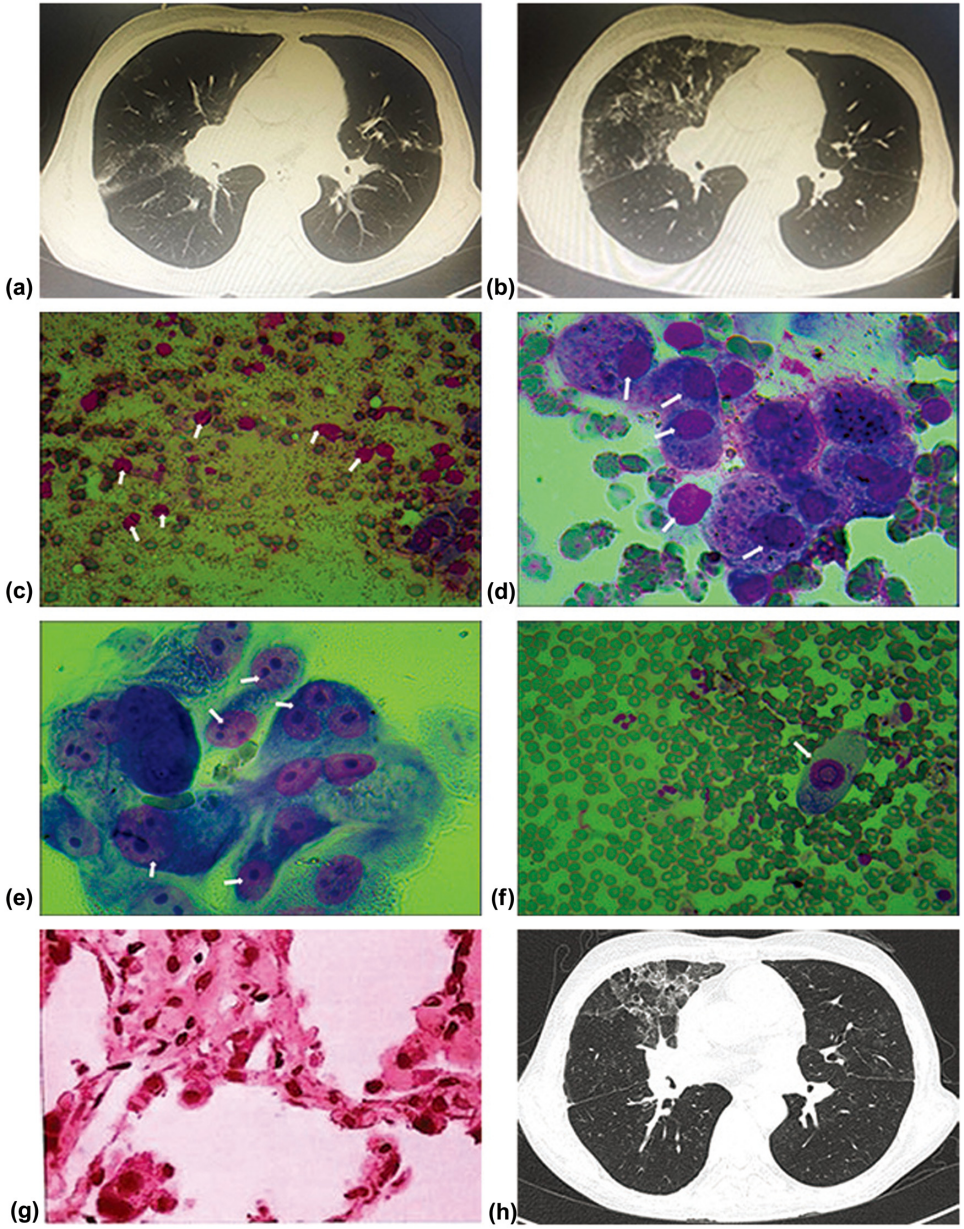
CT, ROSE, and histologic findings of case 1.

Case 1: a 33-year-old male received bilateral lung transplantation for idiopathic pulmonary hypertension. He was given postoperative ventilatory support for 10 days. 251 days post-transplant, patients presented with fever, dyspnea, cough, and sputum production. A chest CT scan demonstrated ground-glass shadows in the right upper and middle lobe (Figure 1a). *Pneumocystis carinii* pneumonia (PCP) was suspected based on clinical presentation and chest CT scan and empiric therapy including caspofungin, cotrimoxazole, and methylprednisolone were initiated based on clinical diagnosis. There was no clinical response to this treatment, and continuous monitoring of chest CT showed lesions progressed on imaging. There were new-onset ground-glass shadows in the right middle (Figure 1b). Bronchoscopy was performed, coincident with sending BALF and TBLB samples for next-generation sequencing, ROSE was performed. Infiltration of lymphocytes (Figure 1c), some macrophages (Figure 1d), and a large number of fibroblasts (Figure 1e) were observed by ROSE. What’s more, the owl-eye sign was observed, which is a sign of what is biologically considered the intranuclear inclusion body of the virus. Figure 1f shows representative microphotographs of CMV infection detected using ROSE. Within 48 h after receipt of the samples, next-generation sequencing analysis detected sequence reads corresponding to CMV infection both in the patient’s BALF and TBLB samples. Therefore, caspofungin, sulfamethoxazole was discontinued and methylprednisolone was tapered, and antibiotic coverage was narrowed to intravenous ganciclovir. After 7 days of receiving TBLB samples, CMV infection was detected on transbronchial biopsy by immunohistochemistry (Figure 1g). The patient gradually recovered with the resolution of ground-glass shadows as observed on serial CT scans (Figure 1h).

**Figure 2:**
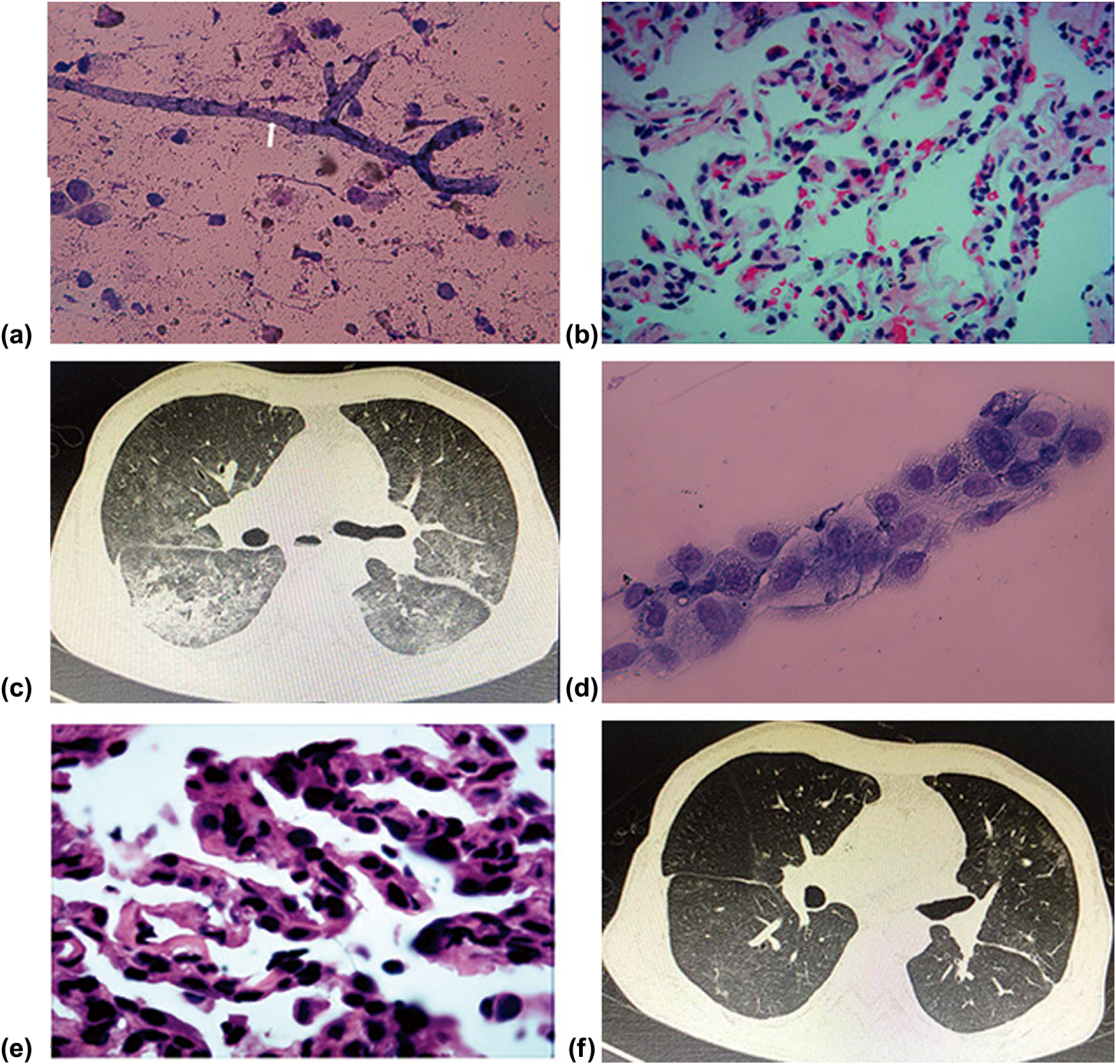
CT, ROSE, and histologic findings of case 2 and 3.

Case 2: a 38-year-old male received bilateral lung transplantation for idiopathic pleuroparenchymal fibroelastosis. He received prophylaxis against virus infection with intravenous ganciclovir and bacteria with broad-spectrum antibiotics. About 599 days post-transplant, he was admitted with dyspnea, cough, and sputum. Bronchoscopy revealed that purulent secretion was attached to bronchial walls. BAL and TBLB were performed under bronchoscopy. ROSE was performed and typical septate hyphae with dichotomous branches were observed by ROSE. So the diagnosis of aspergillosis was made with ROSE (Figure 2a) and mNGS. Routine histologic examination failed to find typical septate hyphae and had negative hexamine silver staining, which showed scattered and infrequent mononuclear cells, primarily lymphocytes, and infiltrates in alveolar tissues (Figure 2b). The pulmonary fungal disease was treated with voriconazole and reduction of immunosuppression with a good response after treatment.

Case 3: a 20-year-old male received bilateral lung transplantation for Graft-versus-Host disease. About 314 days post-transplant, he was admitted with dyspnea and anorexia. CT scan demonstrated ground-glass shadows in bilateral lungs (Figure 2c). Bronchoscopy was performed, BALF and TBLB were obtained under bronchoscopy. ROSE was performed, coincident with sending BALF and TBLB samples for next-generation sequencing. Type II epithelial cell hyperplasia and infiltration of macrophages were noted by ROSE (Figure 2d). Within 48 h after receipt of the samples, mNGS detected sequence reads corresponding to PCP both in the patient’s BALF and TBLB samples. Routine histologic examination demonstrated that there were lymphocytes and scattered neutrophils in alveolar tissues (Figure 2e). The patient received treatment against *Pneumocystis carinii* with caspofungin, cotrimoxazole, and methylprednisolone. Two weeks later, the patient recovered with the resolution of ground-glass shadows as observed on serial CT scans (Figure 2f).

**Figure 3:**
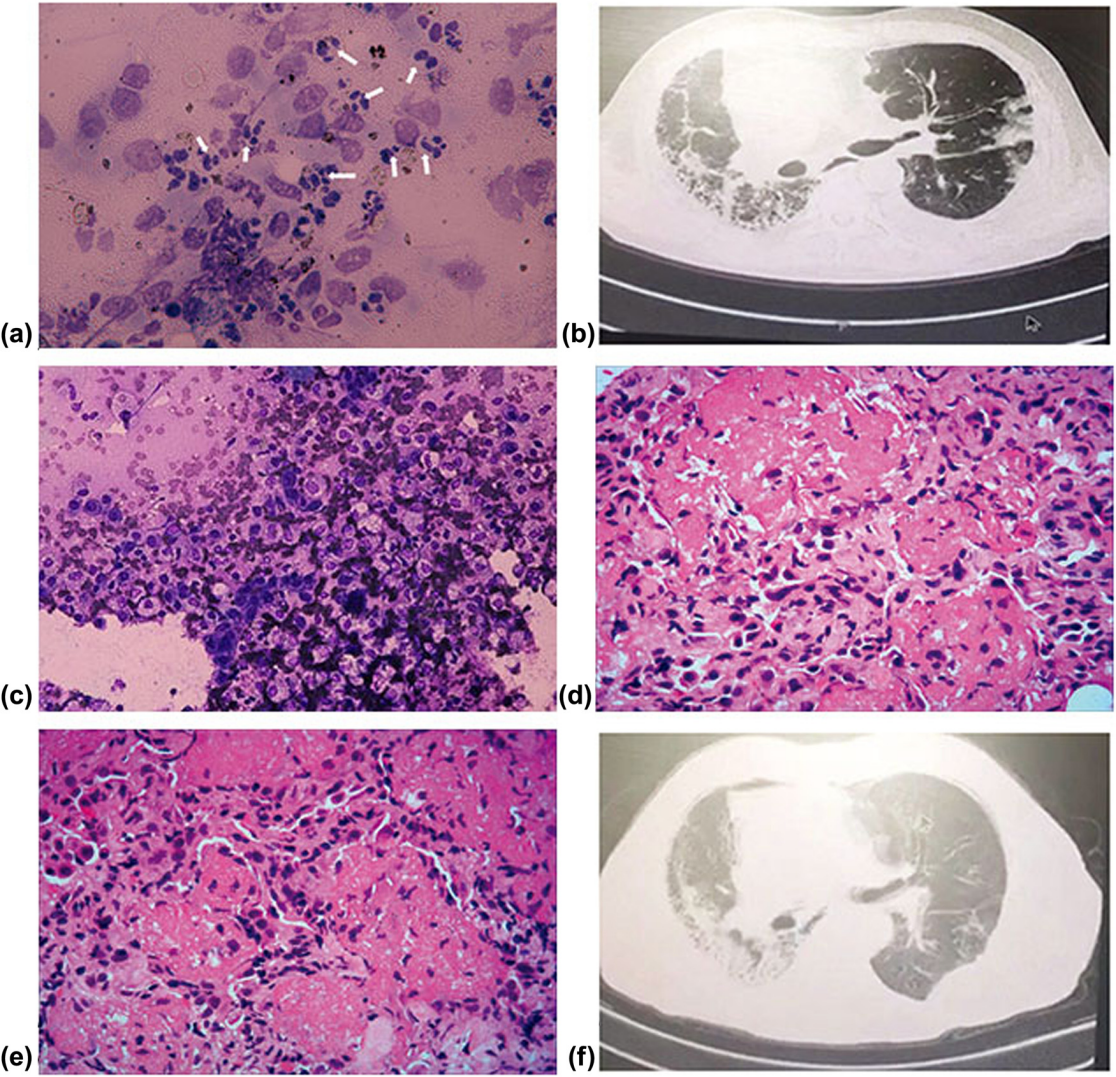
CT, ROSE, and histologic findings of case 4 and 5.

Case 4: a 52-year-old male received a right single lung transplant for IPF. He received prophylaxis against viral and fungal infections. About 45 days post-transplant, he was admitted with dyspnea cough and sputum. CT scan demonstrated consolidations in the right lower lobe. Bronchoscopy was performed, and he was diagnosed with anastomotic stenoses and treated with bronchoscopic balloon dilatation. TBLB was obtained under bronchoscope, which was evaluated by ROSE. A large number of neutrophils were observed on cytologic glass slides under light microscopy (Figure 3a). mNGS was positive for bacterial. This patient recovered after treatment with broad-spectrum antibiotics against bacterial.

Case 5: a 56-year-old male received a left single lung transplant for IPF. About 74 days post-transplant, he was admitted with dyspnea, cough, and fever. CT scan demonstrated consolidation and ground-glass opacification in the left upper and lower lobe (Figure 3b), Bronchoscopy was performed, coincident with sending BAL and TBLB samples for mNGS, ROSE was performed. Infiltration of lymphoid cells and a large number of foamed macrophages and fibroblasts were observed by ROSE (Figure 3c). Lung biopsy specimens were fixed and embedded for routine histologic examination. mNGS didn’t detect clinically significant pathogens both in the patient’s BALF and TBLB samples. TBLB biopsy reveals acute fibrinoid organizing pneumonia (AFOP) which was characterized by open bronchioles with a peribronchiolar deposition of intra-alveolar loose fibrillary fibrin filling the alveolar space with minimal inflammatory infiltrate or interstitial thickening (Figure 3d and e). The patient’s symptom improved with pulse methylprednisolone, with a resolution of consolidation and ground-glass opacification as observed on serial CT scans (Figure 3f).

**Figure 4:**
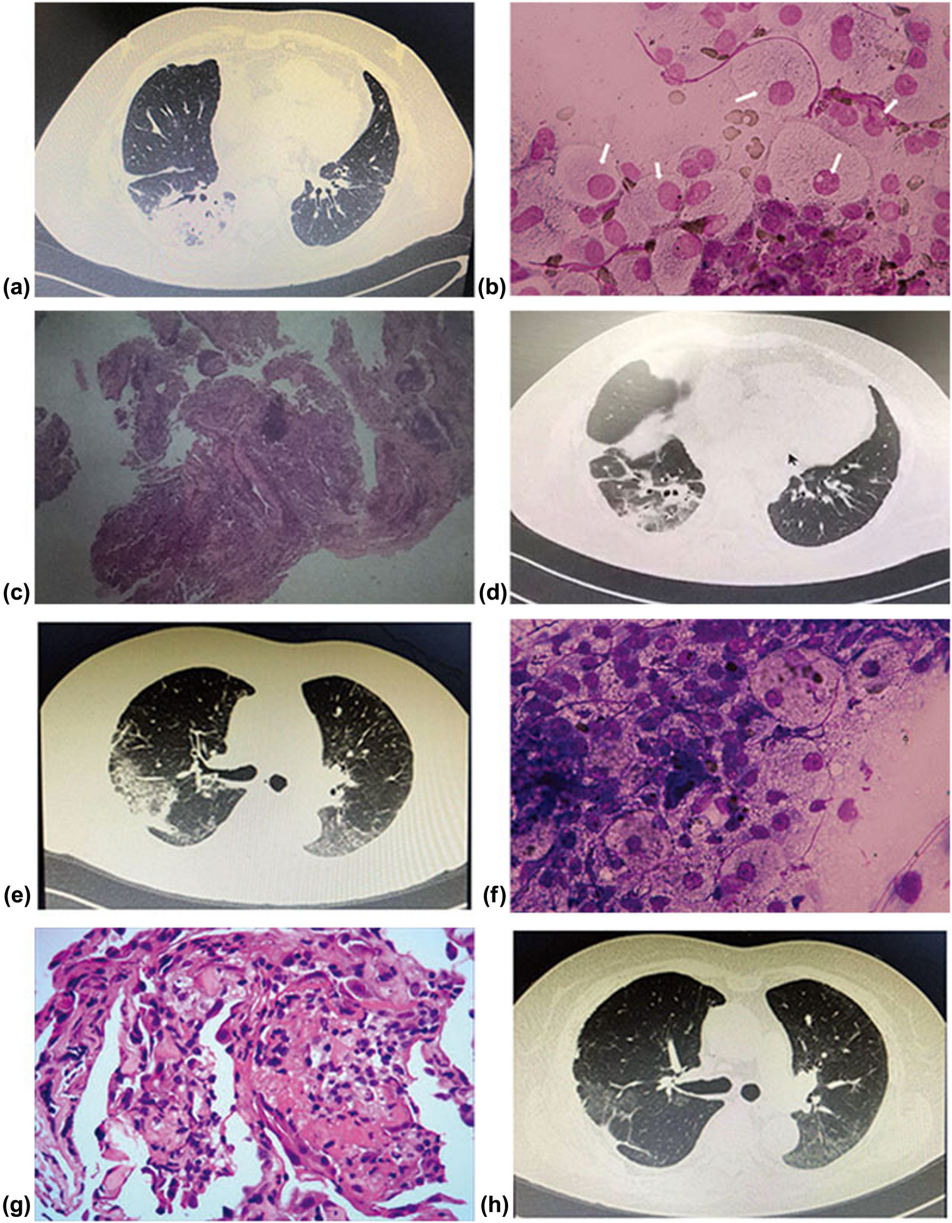
CT, ROSE, and histologic findings of case 6 and 7.

Case 6: a 45-year-old male received bilateral lung transplantation for congenital bronchiectasis. The early postoperative course was unremarkable. About 373 days post-transplant, the patient presented with dyspnea without fever cough and sputum. CT scan demonstrated consolidation in the right lower lobe (Figure 4a). Bronchoscopy was performed, coincident with sending BALF samples for mNGS, ROSE was performed. Infiltration of lymphoid cells and a large number of foamed macrophages (Figure 4b) were observed by ROSE, which conformed to organizing pneumonia. mNGS didn’t detect the clinically significant pathogens in the patient’s BALF. After 10 days of receiving TBLB samples, TBLB biopsy reveals organizing pneumonia (Figure 4c). The patient’s symptoms improved with methylprednisolone, with a partial resolution of consolidation as observed on serial CT scans one month after treatment (Figure 4d).

Case 7: a 73-year-old female received a right single lung transplant for secondary pulmonary interstitial fibrosis. she was given postoperative ventilatory support for 3 days. About 126 days post-transplant, she was admitted with dyspnea on exertion. CT scan demonstrated consolidation and ground-glass opacification in the right upper (Figure 4e) and middle lobe. Bronchoscopy was performed, ROSE of lung biopsy specimens was performed. Infiltration of lymphoid cells and macrophages (Figure 4f) were observed by ROSE. TBLB biopsy reveals minimal acute rejection, there were scattered and infrequent mononuclear cells, primarily lymphocytes, infiltrating in the adjacent interstitium. There were also a few intra-alveolar loose fibrillary fibrin filling the alveolar space (Figure 4g). The patient was treated with increased immunosuppression, the clinical symptoms and radiographic abnormalities were remarkably improved. High-resolution CT findings are shown in Figure 4h.

## 4 Discussions

Conventional microbiologic diagnostic procedures often fail to identify the etiology of lower respiratory tract infections in transplant recipients. Currently, conventional microbiologic diagnosis tests including serologic tests, special microorganism staining, and cultures are routinely employed to identify viral, bacterial, and fungal pathogens. The conventional culture has several drawbacks: first, culture is time-consuming, results from cultures may require up to 72 h; second, the organism detection rate of conventional culture is low due to the early administration of prophylactic antimicrobial drugs, as well as organisms infecting the immunocompromised host can be fastidious to grow or non-cultivable [12,13,14]; third, the spectrum of available assay microbes is limited because the culture is employed by using selective culture media designed for specific pathogens, the culture condition is necessarily biased toward known previously encountered pathogens whereas novel, slow-growing, or rare microbes. The intrinsic limitations of microbiologic tests in terms of speed, sensitivity, and spectrum of available assay microbes [15] makes the early and precise determination of etiologic pathogens challenging in most patients. Consequently, empiric antibiotic therapy including two or three broad-spectrum agents is prescribed for 7 or more days. This “one-size-fits-all” practice is hazardous for individual patients, who may receive insufficient or disproportionately intense antibiotics. In this study, four cases with pulmonary infection were negative for routine culture. In case one, PCP was suspected based on clinical presentation and chest CT scan, and empiric therapy was initiated at the beginning. Fortunately, there is no threat to the patient’s life. Because we accurately detect etiologic pathogens by using mNGS after treatment failure, which guide optimal antimicrobial treatment. If we try to detect etiologic pathogens as early as possible at the beginning, we can take fewer detours.

These deficiencies of conventional culture have led to an effort to develop new methods for the rapid identification of etiologic pathogens, which is essential for targeted antimicrobial treatments. We have developed a new concept, TBLB + ROSE + mNGS, to timely distinguish infection and rejection, and accurately detect etiologic pathogens. ROSE is used to ensure that adequate and diagnostic specimen are harvested by TBLB, then mNGS could accurately detect etiologic pathogens using the biopsy specimens.

ROSE involves the immediate assessment of cytology during the examination. The smears are stained by Diff-Quik stain very quickly (approximately 1 min) and evaluated under light microscopy by the cytotechnologist for sample adequacy and preliminary diagnosis. The bronchoscopist modified or terminated the sampling process based on the information provided by the cytotechnologist, which ensured that the diagnostic material was harvested and ensured sampling of adequate material for appropriate triage of the sample for immunohistochemistry or microbiology studies. What’s more, ROSE is also helpful in providing a preliminary diagnosis, which allows treatment to be initiated earlier. Just like we reported cases in the present article: in case 1, the intranuclear inclusion body of the virus, also called the owl-eye sign, was observed by ROSE which is a sign of virus infection; in case 2, infiltration of inflammatory cells and fungal hyphae from aspergillus was observed; in case 4, a large number of neutrophils were observed, certainly, there are other causes of neutrophilic airways changes in lung transplantation, including the well-described neutrophilic reversible airways dysfunction. After a comprehensive analysis of clinical symptoms, ROSE performance, and mNGS results, the patient was diagnosed with a bacterial infection. The patient recovered after treatment with broad-spectrum antibiotics against bacterial. These examples show the roles of ROSE in early recognition of infection. We also tried to explore the findings of ROSE of several types of rejection just as we report cases in this article. In case 6, infiltration of lymphoid cells and a large number of foamed macrophages were observed by ROSE, which are the cytological features of organizing pneumonia. Histologically, it is defined by the presence of buds of granulation tissue (Masson bodies) in alveoli and alveolar ducts. In case 7, the infiltration of lymphoid cells and some macrophages were observed by ROSE. TBLB biopsy cytopathological study reveals minimal acute rejection, scattered and infrequent mononuclear cells, primarily lymphocytes, infiltrating in the adjacent interstitium. According to our experience, the diagnosis at ROSE correlates well with the final cytopathological diagnosis.

Once we suspected special pulmonary infection based on the clinical, CT scan, and ROSE or the patient received empiric treatment but with poor effect. TBLB samples are collected and assessed by ROSE to ensure sampling of adequate material and then sent for mNGS to search for the infectious pathogens. mNGS allows for the detection of virtually infectious microorganisms present in a given sample based on unbiased sequence analyses [9,10]. Since mNGS was introduced for routine diagnostics in 2014, mNGS has been applied for the identification of bacteria from the blood of septic patients [16], urine, vaginal swabs, or sputum [17,18,19,20] and identification of *Leptospira* from cerebrospinal fluid [21,22,23,24,25,26]. A recent paper, by De Vlaminck et al. [27], reports the identification of predominantly viral pathogens in cell-free DNA in plasma from patients after lung transplantation. In this article, within 48 h after receipt of the samples, mNGS detected etiologic microorganisms missed by conventional cultures.

mNGS has several advantages compared with conventional cultures: first, mNGS has potential benefits in speed, the turnaround time is less than 48 h. In contrast, the average feedback time of pathogen culture is ≥3 days for bacteria, 7 days for fungi, and 45 days for mycobacteria [28]. Second, mNGS has potential benefits in sensitivity, mNGS could yield higher sensitivity for early identification of fastidious microbes (e.g., virus, anaerobe, and fungus) [28]. Third, mNGS can universally cover most medically relevant bacteria, fungi, and virus in a single test, which is a powerful tool both in the detection of known pathogens as well as in identification of unexpected, novel pathogens [29], or fastidious pathogens [30]. What’s more, mNGS yield rate is less likely to be affected by prior antibiotic usage, in contrast with cultures [28,31]. However, there are also challenges in the implementation of mNGS: irst of all, no appropriate thresholds that may indicate a pathogenic infection have been established in the literature so far [32]. Then, mNGS will not distinguish between live and quiescent microorganisms or extracellular DNA from dead microorganisms. Therefore, the application of mNGS in the clinical setting will require thorough knowledge of the patient’s clinical history and require careful analysis of clinical manifestations and the clinical relevance of each organism identified. To the best of our knowledge, this is the first report of using TBLB in conjunction with ROSE and mNGS to differentiate infection and rejection.

There were some limitations to this study. First, because the number of patients was small, statistical analysis was not available. Second, it was a single-center retrospective study. Additional prospective studies with a larger patient cohort are necessary to validate the value of TBLB + ROSE + mNGS in timely distinguishing infection and rejection.

## Abbreviations

AFOP: acute fibrinoid organizing pneumonia
BAL: bronchoalveolar lavage
BALF: bronchoalveolar lavage fluid
CMV: cytomegalovirus
CT: computed tomography
GM test: Galactomannan antigen detection
IPF: idiopathic pulmonary fibrosis
mNGS: metagenomic next-generation sequencing
PCP: *Pneumocystis carinii* pneumonia
ROSE: rapid on-site cytological evaluation
TBLB: transbronchial lung biopsy
X-pert: Mycobacterium tuberculosis/rifampicin resistance test

## References

[1] Whitson BA, Hayes Jr D. Indications and outcomes in adult lung transplantation. J Thorac Dis. 2014;6(8):1018–23. 10.3978/j.issn.2072-1439.2014.07.04. PMC4133539 25132968

[2] Stehlik J, Bavaria JE, Bax J, Cronenwett JL, Edwards LB, Fairman RM, et al. Heart, lung, and vascular registries: evolving goals, successful approaches, and ongoing innovation. J Heart Lung Transplant. 2016 Oct;35(10):1149–57. 10.1016/j.healun.2016.08.021. 27772667

[3] Yusen RD, Edwards LB, Dipchand AI, Goldfarb SB, Kucheryavaya AY, Levvey BJ, et al. International Society for Heart and Lung Transplantation. The Registry of the International Society for Heart and Lung Transplantation: Thirty-third Adult Lung and Heart-Lung Transplant Report-2016; Focus Theme: Primary Diagnostic Indications for Transplant. J Heart Lung Transplant. 2016 Oct;35(10):1170–84. 10.1016/j.healun.2016.09.001. Epub 2016 Sep 13. 27772669

[4] Yusen RD, Edwards LB, Kucheryavaya AY, Benden C, Dipchand AI, Goldfarb SB, et al. The Registry of the International Society for Heart and Lung Transplantation: thirty-second Official Adult Lung and Heart-Lung Transplantation Report – 2015; focus theme: early graft failure. J Heart Lung Transplant. 2015 Oct;34(10):1264–77. 10.1016/j.healun.2015.08.014. 26454740

[5] De Vito Dabbs A, Hoffman LA, Iacono AT, Zullo TG, McCurry KR, Dauber JH. Are symptom reports useful for differentiating between acute rejection and pulmonary infection after lung transplantation?Heart Lung. 2004;33(6):372–80. 10.1016/j.hrtlng.2004.05.001. 15597291

[6] Roden AC, Aisner DL, Allen TC, Aubry MC, Barrios RJ, Beasley MB, et al. Diagnosis of acute cellular rejection and antibody-mediated rejection on lung transplant biopsies: a perspective from members of the pulmonary pathology society. Arch Pathol Lab Med. 2017 Mar;141(3):437–44. 10.5858/arpa.2016-0459-SA. Epub 2016 Nov 7. PMID: 27819763. 27819763

[7] Liu N, Kan J, Cao W, Cao J, Jiang E, Zhou Y, et al. Metagenomic next-generation sequencing diagnosis of peripheral pulmonary infectious lesions through virtual navigation, radial EBUS, ultrathin bronchoscopy, and ROSE. J Int Med Res. 2019 Oct;47(10):4878–85. 10.1177/0300060519866953. Epub 2019 Aug 22. PMID: 31436107; PMCID: PMC6833387. PMC6833387 31436107

[8] Huang J, Jiang E, Yang D, Wei J, Zhao M, Feng J, et al. Metagenomic next-generation sequencing versus traditional pathogen detection in the diagnosis of peripheral pulmonary infectious lesions. Infect Drug Resist. 2020 Feb 19;13:567–76. 10.2147/IDR.S235182. PMID: 32110067; PMCID: PMC7036976. PMC7036976 32110067

[9] Victoria JG, Kapoor A, Li L, Blinkova O, Slikas B, Wang C, et al. Metagenomic analyses of viruses in stool samples from children with acute flaccid paralysis. J Virol. 2009 May;83(9):4642–51. 10.1128/JVI.02301-08. PMC2668503 19211756

[10] Mokili JL, Rohwer F, Dutilh BE. Metagenomics and future perspectives in virus discovery. Curr Opin Virol. 2012 Feb;2(1):63–77. 10.1016/j.coviro.2011.12.004. PMC7102772 22440968

[11] Stewart S, Fishbein MC, Snell GI, Berry GJ, Boehler A, Burke MM, et al. Revision of the 1996 working formulation for the standardization of nomenclature in the diagnosis of lung rejection. J Heart Lung Transplant. 2007 Dec;26(12):1229–42. 10.1016/j.healun.2007.10.017. PMID: 18096473. 18096473

[12] Kitsios GD, Fitch A, Manatakis DV, Rapport SF, Li K, Qin S, et al. Respiratory microbiome profiling for etiologic diagnosis of pneumonia in mechanically ventilated patients. Front Microbiol. 2018 Jul 10;9:1413. 10.3389/fmicb.2018.01413. PMC6048198 30042738

[13] Spoelstra-de Man AM, Girbes AR. Comment on “Surviving Sepsis Campaign: International guidelines for management of severe sepsis and septic shock: 2008” by Dellinger et al. Intensive Care Med. 2008 Jun;34(6):1160–2; author reply 1163-4. 10.1007/s00134-008-1089-5. PMC2480487 18415078

[14] Fenollar F, Raoult D. Molecular diagnosis of bloodstream infections caused by non-cultivable bacteria. Int J Antimicrob Agents. 2007 Nov;30(Suppl 1):S7–15. 10.1016/j.ijantimicag.2007.06.024 17707613

[15] Jain S, Self WH, Wunderink RG, Fakhran S, Balk R, Bramley AM, et al. Commnity-acquired pneumonia requiring hospitalization among U.S. adults. N Engl J Med. 2015 Jul 30;373(5):415–27. 10.1056/NEJMoa1500245. PMC4728150 26172429

[16] Grumaz S, Stevens P, Grumaz C, Decker SO, Weigand MA, Hofer S, et al. Next-generation sequencing diagnostics of bacteremia in septic patients. Genome Med. 2016 Jul 1;8(1):73. 10.1186/s13073-016-0326-8. PMC4930583 27368373

[17] Seth-Smith HM, Harris SR, Skilton RJ, Radebe FM, Golparian D, Shipitsyna E, et al. Whole-genome sequences of Chlamydia trachomatis directly from clinical samples without culture. Genome Res. 2013 May;23(5):855–66. 10.1101/gr.150037.112. PMC3638141 23525359

[18] Doughty EL, Sergeant MJ, Adetifa I, Antonio M, Pallen MJ. Culture-independent detection and characterisation of Mycobacterium tuberculosis and M. africanum in sputum samples using shotgun metagenomics on a benchtop sequencer. PeerJ. 2014 Sep 23;2:e585. 10.7717/peerj.585. PMC4179564 25279265

[19] Andersson P, Klein M, Lilliebridge RA, Giffard PM. Sequences of multiple bacterial genomes and a Chlamydia trachomatis genotype from direct sequencing of DNA derived from a vaginal swab diagnostic specimen. Clin Microbiol Infect. 2013 Sep;19(9):E405–8. 10.1111/1469-0691.12237. 23647919

[20] Hasman H, Saputra D, Sicheritz-Ponten T, Lund O, Svendsen CA, Frimodt-Møller N, et al. Rapid whole-genome sequencing for detection and characterization of microorganisms directly from clinical samples. J Clin Microbiol. 2014 Jan;52(1):139–46. 10.1128/JCM.02452-13. Epub 2013 Oct 30. Erratum in: J Clin Microbiol. 2014 Aug;52(8):3136. PMC3911411 24172157

[21] Wilson MR, Naccache SN, Samayoa E, Biagtan M, Bashir H, Yu G, et al. Actionable diagnosis of neuroleptospirosis by next-generation sequencing. N Engl J Med. 2014 Jun 19;370(25):2408–17. 10.1056/NEJMoa1401268. PMC4134948 24896819

[22] Be NA, Allen JE, Brown TS, Gardner SN, McLoughlin KS, Forsberg JA, et al. Microbial profiling of combat wound infection through detection microarray and next-generation sequencing. J Clin Microbiol. 2014 Jul;52(7):2583–94. 10.1128/JCM.00556-14. PMC4097755 24829242

[23] Brown JR, Morfopoulou S, Hubb J, Emmett WA, Ip W, Shah D, et al. Astrovirus VA1/HMO-C: an increasingly recognized neurotropic pathogen in immunocompromised patients. Clin Infect Dis. 2015 Mar 15;60(6):881–8. 10.1093/cid/ciu940. PMC4345817 25572899

[24] Kommedal Ø, Wilhelmsen MT, Skrede S, Meisal R, Jakovljev A, Gaustad P, et al. Massive parallel sequencing provides new perspectives on bacterial brain abscesses. J Clin Microbiol. 2014 Jun;52(6):1990–7. 10.1128/JCM.00346-14. PMC4042731 24671797

[25] Naccache SN, Peggs KS, Mattes FM, Phadke R, Garson JA, Grant P, et al. Diagnosis of neuroinvasive astrovirus infection in an immunocompromised adult with encephalitis by unbiased next-generation sequencing. Clin Infect Dis. 2015 Mar 15;60(6):919–23. 10.1093/cid/ciu912. PMC4345816 25572898

[26] Naccache SN, Federman S, Veeraraghavan N, Zaharia M, Lee D, Samayoa E, et al. A cloud-compatible bioinformatics pipeline for ultrarapid pathogen identification from next-generation sequencing of clinical samples. Genome Res. 2014 Jul;24(7):1180–92. 10.1101/gr.171934.113. PMC4079973 24899342

[27] De Vlaminck I, Martin L, Kertesz M, Patel K, Kowarsky M, Strehl C, et al. Noninvasive monitoring of infection and rejection after lung transplantation. Proc Natl Acad Sci U S A. 2015 Oct 27;112(43):13336–41. 10.1073/pnas.1517494112. PMC4629384 26460048

[28] Miao Q, Ma Y, Wang Q, Pan J, Zhang Y, Jin W, et al. Microbiological diagnostic performance of metagenomic next-generation sequencing when applied to clinical practice. Clin Infect Dis. 2018 Nov 13;67(Suppl 2):S231–40. 10.1093/cid/ciy693. 30423048

[29] Zaki AM, van Boheemen S, Bestebroer TM, Osterhaus AD, Fouchier RA. Isolation of a novel coronavirus from a man with pneumonia in Saudi Arabia. N Engl J Med. 2012 Nov 8;367(19):1814–20. 10.1056/NEJMoa1211721. 23075143

[30] Li H, Gao H, Meng H, Wang Q, Li S, Chen H, et al. Detection of pulmonary infectious pathogens from lung biopsy tissues by metagenomic next-generation sequencing. Front Cell Infect Microbiol. 2018 Jun 25;8:205. 10.3389/fcimb.2018.00205. eCollection 2018. PubMed PMID: 29988504; PubMed Central PMCID: PMC6026637. PMC6026637 29988504

[31] Rhodes J, Hyder JA, Peruski LF, Fisher C, Jorakate P, Kaewpan A, et al. Antibiotic use in Thailand: quantifying impact on blood culture yield and estimates of pneumococcal bacteremia incidence. Am J Trop Med Hyg. 2010 Aug;83(2):301–6. 10.4269/ajtmh.2010.09-0584. PMC2911175 20682872

[32] Petty TJ, Cordey S, Padioleau I, Docquier M, Turin L, Preynat-Seauve O, et al. Comprehensive human virus screening using high-throughput sequencing with a user-friendly representation of bioinformatics analysis: a pilot study. J Clin Microbiol. 2014 Sep;52(9):3351–61. 10.1128/JCM.01389-14. PMC4313162 25009045

